# Digi-Do: a digital information tool to support patients with breast cancer before, during, and after start of radiotherapy treatment: an RCT study protocol

**DOI:** 10.1186/s12911-021-01448-3

**Published:** 2021-02-25

**Authors:** Sofi Fristedt, Frida Smith, Annika Grynne, Maria Browall

**Affiliations:** 1 Jönköping Academy For Improvement of Health and Welfare and IMPROVE, School of Health and Welfare, Jönköping University, Jönköping, Sweden; 2 Department of Health Sciences, Faculty of Medicine, Lund University, Lund, Sweden; 3 Regional Cancer Centre West, Gothenburg, Sweden; 4 Department of Technology Management and Economics, Chalmers University of Technology, Gothenburg, Sweden; 5 Department of Nursing and IMPROVE, School of Health and Welfare, Jönköping University, Jönköping, Sweden; 6 Affiliated with the Department of Oncology, Institute of Clinical Sciences, Sahlgrenska Academy, University of Gothenburg, Gothenburg, Sweden

**Keywords:** Evaluation, Health literacy, Participatory design, Self-efficacy, Virtual reality

## Abstract

**Background:**

Radiation Therapy (RT) is a common treatment after breast cancer surgery and a complex process using high energy X-rays to eradicate cancer cells, important in reducing the risk of local recurrence. The high-tech environment and unfamiliar nature of RT can affect the patient’s experience of the treatment. Misconceptions or lack of knowledge about RT processes can increase levels of anxiety and enhance feelings of being unprepared at the beginning of treatment. Moreover, the waiting time is often quite long. The primary aim of this study will be to evaluate whether a digital information tool with VR-technology and preparatory information can decrease distress as well as enhance the self-efficacy and health literacy of patients affected by breast cancer before, during, and after RT. A secondary aim will be to explore whether the digital information tool increase patient flow while maintaining or increasing the quality of care.

**Method:**

The study is a prospective and longitudinal RCT study with an Action Research participatory design approach including mixed-methods data collection, i.e., standardised instruments, qualitative interviews (face-to-face and telephone) with a phenomenological hermeneutical approach, diaries, observations, and time measurements, and scheduled to take place from autumn 2020 to spring 2022. The intervention group (n = 80), will receive standard care and information (oral and written) and the digital information tool; and the control group (n = 80), will receive standard care and information (oral and written). Study recruitment and randomisation will be completed at two centres in the west of Sweden.

**Discussion:**

Research in this area is scarce and, to our knowledge, only few previous studies examine VR as a tool for increasing preparedness for patients with breast cancer about to undergo RT that also includes follow-ups six months after completed treatment. The participatory approach and design will safeguard the possibilities to capture the patient perspective throughout the development process, and the RCT design supports high research quality. Digitalisation brings new possibilities to provide safe, person-centred information that also displays a realistic picture of RT treatment and its contexts. The planned study will generate generalisable knowledge of relevance in similar health care contexts.

*Trial registration*: ClinicalTrials.gov Identifier: NCT04394325. Registered May 19, 2020. Prospectively registered.

## Background

Among women, breast cancer is the most diagnosed cancer and the leading cause of cancer death worldwide [1]. In 2019, the Swedish national cancer register recorded 9225 cases of breast cancer; 9157 women and 68 men (0.7%). The incidence rate of breast cancer in Sweden is expected to continue to rise, due to an increase in both the general and ageing population numbers [2]. Radiation Therapy (RT) is a common treatment (received by 75–80% of breast cancer patients) after surgery and is a complex process based on the use of high energy X-rays to eradicate cancer cells, important in reducing the risk of local recurrence [1]. While the RT procedure itself takes only a few minutes and is painless, it requires meticulous preparation and patients then need to attend the RT treatment centre 5 days a week for up to 3–5 weeks. Moreover, in Sweden, the waiting time is often quite long, sometimes up to 10 weeks [2]. The high-tech environment and unfamiliar nature of RT can affect the patient’s experience of the treatment [3–5]. Furthermore, misconceptions or a lack of knowledge about RT processes can increase their levels of anxiety and enhance feelings of being unprepared at the beginning of treatment [6–8].

Having received correct information about future treatment may enable patients to realistically assess advantages and disadvantages as well as foresee possible treatment results. This may in turn create a feeling of increased knowledge and security as well as reduce distress for the patient [9–13]. A number of Swedish acts state that the healthcare system must provide each patient with individually tailored information and the opportunity to choose between different treatments [14–16] or stipulate that healthcare providers should actively ensure that the information has been received and understood by the patient [10]. Having a person-centred approach to information provision and communication means to enhance shared decision making, seeing the person as a capable individual who wants to adapt generic information to his or her social context and experiences [17, 18].

When informing patients, their health literacy must also be considered, i.e., their ability to acquire, understand, and use information about their health, including that related to cognitive and social functions [19]. Health literacy can be seen as being dynamic, and hence can fluctuate, depending on the state of the individual, the situation, the culture, or the environment [20].

Self-efficacy, referring to a persons’ belief in his/her ability to deal with specific situations [21] such as in this case a cancer diagnosis, treatments, and transitioning to survivorship [22]. Cancer patients’ perceived self‐efficacy may directly or indirectly affect health behaviours, lifestyle adjustments and psychological growth, psychological distress, and physical outcomes, as well as quality of life (QoL) [23, 24]. Psychosocial distress may appear early in the diagnostic process and can have negative effects on compliance with treatment and subsequent quality of life [22].

There is a need for new ways to inform and involve patients, hence there is a strong call for digitalized tools to be used in healthcare. To be effective, however, digital tools must be developed based on research that also follows quality standards [25]. More and more, patients request they are provided information through videos, accessible for later review at home and for sharing with loved ones, as many patients travel substantial distances to receive care [26]. Furthermore, videos and audio-visual materials have proven to be successful communication tools and the format also supports the distribution and sharing of information with others [27–29]. Virtual reality (VR) has the inherent potential to be interactive because its content is generated in real time, not ahead of time, which makes the experience even more immersive [30]. Specifically, VR differs from other forms of media because it induces the sense of “presence”, i.e., the feeling of “being there” inside the virtual experience produced by the technology [31]. VR-interventions for cancer patients were investigated in a meta-analysis by Zeng et al. [32], showing its effects on improved learning through cognitive training and significantly decreased fatigue. A recent study indicated that a virtual reality education for breast cancer patients could improve RT knowledge and perhaps decrease anxiety as well as was appreciated by the patients [12]. Moreover, virtual reality radiotherapy educational program could have a positive effect on patients prior to their initial RT session by providing them with useful content and decreasing their anxiety about the process, thus increasing the patient RT comprehension [33].

In another study, the majority (86%) of the participants considered that the information they received did not provide them with an adequate understanding of the treatment and what it entailed [34]. Participants identified potential benefits of VR video viewing before the first day of treatment and felt that it could increase understanding of the treatment process, specifically the spatial and acoustic aspects of treatment. The authors concluded that a VR education tool has the potential to enhance standard patient education, increasing understanding of treatment and decreasing anxiety.

However, the potential outcomes related to Health Literacy are scarcely evaluated. Moreover, most studies were of relatively low methodological quality or pilot studies and the need for research of more robust study design and larger sample sizes has been identified.

The primary aim of this study will be to evaluate whether a digital information tool with VR-technology and preparatory information can decrease distress as well as enhance the self-efficacy and health literacy of patients affected by breast cancer before, during, and after RT. A secondary aim will be to explore whether the digital information tool increase patient flow while maintaining or increasing the quality of care.

## Methods

### Study design

The study (ClinicalTrials.gov Identifier: NCT04394325) is a prospective and longitudinal RCT study with an Action Research (AR) participatory design approach, including mixed-method data collection, i.e., standardised instruments, interviews (face-to-face and telephone), diaries, observation, and time measurements. To ensure transparency and rigour the CONSORT [35] checklist for randomised trials and COREQ [36] checklist for qualitive research will be applied. The timeline for the study is scheduled between autumn 2020 to spring 2022. The study will consist of two arms: (A) an intervention group (*n* = 80), who will receive standard care and information and the digital information tool; and (B) a control group (*n* = 80) who will receive standard care and information (oral and written). Recruitment and randomisation will be completed at two hospitals in the Western part of Sweden. Two nurses will act as research assistants at the respective hospitals.

### Sample

Adult patients (> 18 years) diagnosed with breast cancer, stage I–II, who are not receiving adjuvant or neo-adjuvant chemotherapy or anti-Her-2 treatment and with RT as additional treatment after surgery, will be consecutively included during the planned post-operative visit to the surgical clinic. They will also need the ability to read, write, and understand Swedish to adhere to the digital information tool and the data collection instruments, and have access to a smartphone or a tablet. Patients responding to these inclusion criteria will be consecutively asked to participate at the post-surgery follow-up visit and receive information (oral and written) about the study. After providing their signed informed consent, participants will be randomized to either the intervention group (A) or the control group (B) by drawing a note (marked with group A or B) for each participant from a black box. Randomization will be stratified by hospital, and a total of 80 participants will be finally included at each hospital (40 in each group). The participants in the intervention group will receive oral and written information regarding the digital information tool.

#### Sample size and hypothesis

The Distress Thermometer will be used as the primary outcome measure and used as a basis for the power calculation. A sample size of 63 persons in each group (a total of *n* = 126) would give an 85% power to detect a 0.10 improvement in the intervention group at an alpha = 0.05.

The null hypothesis is that there is no difference in distress between the intervention group and the control group; the alternative hypothesis is that the intervention group will experience 10% lower distress than the control group at the follow-up, six months post-completion of RT. The anticipated recruitment rate is 4.4 participants per site and month.

### Development and testing of the digital information tool (Digi-Do)

As a pre-phase, a pilot study was performed for the development and testing of the digital information tool (Digi-Do), approved by the Regional Ethics Committee (Dnr 917-17) with 30 patients in total (15 women with breast cancer and 15 men with prostate cancer). The Digi-Do tool was developed through a co-design process, where patients and staff were involved in all design steps; initial exploration of work, discovery process and prototyping [37]. According to the study’s co-design, iterative methodology [38 in manuscript], changes have since been made to the digital information tool and a second version has now been developed for the full RCT. The present study has been approved by the Swedish Ethical Review Authority (Dnr 2020-00170). An ongoing systematic literature review will also guide the present study, and the project is registered in PROSPERO (PROSPERO; 168073).

The digital information tool applied in this project is divided into two separate but coherent applications (apps) for mobile devices: one (VR-app) with a guided tour of the RT-department with a voice-over to describe 360 images to create a sense of actually having visited the department prior to start of RT, and one (information app) containing information obtained through the pre-treatment phase. If a VR-effect is not desirable, the patient can complete the simulated study-visit on their mobile phone or tablet as well as present the images in the browser of an integrated media player. Three areas of information are available in the information app: (1) Q&As from the existing written information, presented both in writing and a recorded voice; (2) practical information, such as maps, public transportation options with relevant links to public transport, telephone numbers, and information about possibilities for staying at the patient hotel; and (3) three short animated films about cancer and physical activity during RT.

### Data collection

Socio-demographic data concerning age, marital status, number of persons in household, level of education, working/retired/sick leave, stage of disease, co-morbidities, and treatment will be gathered from the patients and/or their medical charts. The instruments for data collection will be administrated in person or by post at the baseline collection, and by post (including a pre-paid return envelope) on consecutive collections (Table 1). Two reminders will be sent for each data collection, separated two weeks in time if necessary. Data will be coded and manually entered in SPSS. The SPSS database will be stored on a secure digital storage (JU share files) at Jönköping University.

**Table 1:** Data collections at different points in time

Point in time	Data collections
Baseline	Socio-demographic and co-morbidity data Distress (DIS-A); Self-efficacy (GSES); Health literacy (functional, communicative & critical, e-HLQ) Notebook written by 30 patients
Week of commencing RT treatment	Distress (DIS-A) Notebook collected and returned after reading
Within a month of completion of RT treatment	In depth/or telephone interviews with participants
Six months post-completion of RT treatment	Distress (DIS-A); Self-efficacy (GSES); Health literacy (functional, communicative & critical, e-HLQ) Telephone interviews with participants

#### Primary outcome measure

*DIS-A. The NCCN Distress Thermometer* is a brief screening tool for cancer patients to assess psychosocial distress [39], including 34 dichotomous items on the presence of physical, emotional, family, practical, and spiritual problems. A score of 4.5 or higher indicates the patient is distressed and in need of support. The Swedish translation of the DT/PL is consistent with the original English version [40].

#### Secondary outcome measure

*The General Self-efficacy Scale* [41]*, validated into Swedish GSES* [42] is an instrument that asks the persons´ opinion about how he or she can deal with difficulties and challenges in life. It consists of 10 items (eg. “I can always manage to solve difficult problems if I try hard enough”, “I am confident that I could deal efficiently with unexpected events” and “I can usually handle whatever comes my way”) rated on a four-point Likert scale (“not at all true” to “exactly true”), summarized across respondents (range 10–40); higher scores indicate higher self-efficacy.

*The communicative and critical health literacy scale*—*Swedish version*: This instrument consists of five items self-rated on a five-point Likert scale, ranging from “not at all” to “precisely”. The first three items include statements focusing on the capacity for collecting, extracting, and understanding relevant health information. The other two items include statements highlighting capacities to judge the reliability of the information and to apply health information to everyday life. In this study we have added an open-ended question concerning information sources [43, 44].

*The Swedish Functional Health Literacy scale:* This scale comprises five items about persons’ skills in reading and understanding health information. The items can be self-assessed on a five-point Likert scale ranging from ‘never’ to ‘often’. In this study, we have excluded two of the original questions (difficulty reading health information and time to read health information) as these were found to be less relevant in relation to our aim, and we added one open-ended question concerning how to remember information relating to health, illness, and treatment [44].

*The eHealth Literacy Questionnaire (eHLQ)* is a psychometrically robust multidimensional tool designed to be used to understand and evaluate people’s interaction with digital health services [45]. The eHLQ consists of 7 scales capturing various attributes of the users and the intersection between users and technologies. The applied Swedish version of the eHLQ (translated from English) is currently undergoing validation testing (the results will be published during 2020).

*The Self-Administered Comorbidity Questionnaire:* The Swedish version of this scale has been modified from the English version, and is a method to assess comorbidity for clinical and health services research [46].

#### Time measurements and field notes

Ten patients respectively from groups A and B will, during the planned post-operative visit to the surgical clinic, be consequently asked to participate in the time measurement and observational part of the study (*n* = 20). Three of the planned treatments for the first week of RT, and, additionally, one session in the last week of treatment, will be measured based on seven timepoints; patient enters door of RT room, patient lies on table, first image, first field beam on, last field beam on, patient leaves table, and patient leaves RT room. During each RT session, questions asked by patients and staff will be documented as field notes, including what subjects are addressed, who initiates the question, and whether, how, and to where the staff refers the patient for further information. These field notes will be taken into account for validation of the content of the information in the app, but not further analysed.

#### Notebook and in-depth interviews

Approximately 30 patients (in group A) will be strategically asked to participate in the qualitative part of the study. The patients will be asked to make notes in a notebook during the waiting period prior to the start of RT as well as during the RT treatment period. The participants will be encouraged to write reflections with a focus on the support, guidance, and information they receive from healthcare professionals, relatives, and the digital information tool in these notebooks. In the first week of RT treatment, patients will submit their diaries to the researchers. The text in the notebooks will be read and summarized to support the development of in-depth questions for upcoming individual interviews. These individual in-depth and/or telephone interviews will take place on two occasions (1–4 weeks and 6 months post-RT completion) and with at least ten of the patients who have accepted participation and have made regular notes. Questions will include topics such as their experiences of receiving the information through the digital information tool during the waiting time and treatment period, of using the digital information tool, to what extent the digital information has affected their involvement and preparedness for the radiation treatment, as well as their experiences during the time since the end of RT.

### Data analysis

#### Quantitative data

Data will be analysed descriptively taking the level of scale into consideration, i.e., using mean (m) and standard deviation (SD) when relevant, or, otherwise, median (md) and range. Group comparison at each point in time will be completed using relevant parametric or non-parametric methods [47]. *P *values < 0.05 will be considered statistically significant. Generalized linear models [47] will be used to analyse differences between groups and for repeated measures over time for the continuous variables, while nominal (categorical) variables will be tested with chi-square test (exact).

#### Qualitative data

A phenomenological hermeneutical approach, with a focus on the essence and the underlying meaning in the lived experience of receiving RT, will be applied [48]. Narratives, in the form of notebook texts, recorded by the participants, and in-depth interviews that encompasses descriptions and expressions of the person´s lived experiences of the phenomenon will be analysed. The analysis involves three steps [48, 49]: (1) the naïve reading, where the text is read numerous times and the researchers initial understanding of the text is written down. (2) The structural analyses, where the text is broken down into meaning units, that embody information about the phenomenon of interest. (3) The comprehensive understanding, in which the text is considered in its entirety, and the findings from the first and second steps, are brought together with the researchers pre-understanding, and discussed in relation to relevant literature.

## Discussion

Research in this area is scarce and, few other studies have examined VR as a tool for increasing preparedness for patients with breast cancer about to undergo RT that also includes follow-ups up to six months after completed treatment. Previous studies on VR for the same purpose are small e.g. [12], while yet including a control and intervention group. Other studies such as [9] that intends to improve patient education for patient with RT, are not based on VR tools.

The participatory approach and design will safeguard the possibilities to capture the patient perspective throughout the development process, and the RCT design supports high research quality. Because the project utilises rather novel technology, it is clearly important to include patient perspectives to ensure that persons with less digital competence are not deprived of or alienated from the information. Patients diagnosed with breast cancer of different ages will be included. This will likely provide us with a sample representing persons of different experiences and variations in life situations and self-efficacy, as well as in their digital competence and health literacy. Such diversity is important to generate as broad a knowledge base as possible for the design of future research projects with different cancer diagnoses and clinical decision-making in relation to RT. The outcomes of this project will be put to practical use, both during and after the research period. The project will contribute to clinical cancer research and innovation by leading to better informed and better prepared patients. Using the waiting time actively as a part of the process of preparing can make this period more meaningful for the patient.

The digital information tools utilised in the present project are also likely to add value to patients’ next-of-kin, by providing a more concrete picture of the treatment and its context. This brings unique possibilities for an unlimited number of next-of-kin of all ages to better understand what their mother, sister, daughter, friend, etc. is experiencing. This will in turn increase the psychosocial support provided to patients and loved ones, thus strengthening the patients’ position in cancer care. The VR innovation creates opportunities for improving care processes and levelling uneven structures. The project may also improve the efficiency of RT by leading to well-prepared patients. The mobile applications can be regarded as an accessible and convenient platform that enables the person to gain instant knowledge and guidance [50].

Because the participatory design builds on collaboration with patients, as well as staff, it will also generate knowledge and a digital information tool that will be easy to implement in clinical practice in similar settings. Moreover, participatory designs support the three Rs of research; namely, to enhance rigor, assure relevance, and ensure a greater as well as broader reach [51].

## Conclusion

. This study will tell us whether a digital information tool with VR-technology and preparatory information can decrease distress as well as enhance self-efficacy and health literacy of patients with a breast cancer about to receive RT. It will also indicate whether a digital information tool can increase patient flow while maintaining or increasing the quality of care. The planned study will generate generalisable knowledge of relevance in similar health care settings and for patients with other diagnosis of cancer about to receive RT.

## Data Availability

Not applicable.

## Abbreviations

App: Software applications
AR: Action Research
DIS-A: The NCCN Distress Thermometer
eHLQ: The eHealth Literacy Questionnaire
GSES: The Swedish version of the General Self-efficacy Scale
M: Mean
QoL: Quality of Life
RCT: Randomised Control Trial
RT: Radiation Therapy
SD: Standard deviation
VR: Virtual reality
